# Use of *Acheta domesticus* meal as a full soybean substitute in the feeding of slow-growing chicks

**DOI:** 10.1016/j.psj.2023.102503

**Published:** 2023-01-16

**Authors:** Jaime Nieto, Javier Plaza, Javier Lara, José-Alfonso Abecia, Isabel Revilla, Carlos Palacios

**Affiliations:** ⁎ Area of Animal Production, Faculty of Environmental and Agricultural Sciences, University of Salamanca, 37007 Salamanca, Spain; † Animal Husbandry and Animal Health Assistance Educational Program, High-School Torres Villarroel, 37008 Salamanca, Spain; ‡ Institute of Research in Environmental Sciences of Aragón (IUCA), University of Zaragoza, 50013 Zaragoza, Spain; § Area of Food Technology, E.P.S. of Zamora, University of Salamanca, 49022 Zamora, Spain

**Keywords:** *Acheta domesticus*, alternative poultry farming, high-protein source, insect meal, slow-growing chick

## Abstract

Sustainable poultry meat production involves the use of slow-growing chick strains and the utilization of new protein sources as an alternative to the current monopoly of soybean meal. In this scenario, a study was conducted to assess the effect of replacing soybean meal with domestic cricket (*Acheta domesticus*) meal on the developing cycle of slow-growing chicks. To this end, a total of 128 one-day-old male chicks (*Colorield*) were randomly assigned into 16 experimental units, each consisting of 8 chicks, which in turn were grouped into 2 groups fed isoproteic and isoenergetic diets in which the protein source differed: the control group (C) fed soybean meal and the Acheta group (AD) fed *Acheta domesticus* insect meal as the main protein source. Chicks were slaughtered at 95 d of age. Three different diets (F1, F2 and F3) were used for each experimental group according to the nutritional needs of the birds during their growth. The F1 diet (1–29 d) resulted in higher feed and water intake and higher body weight gain for group C, but a lower feed conversion rate. On the contrary, during F2 (29–60 d) no differences in productive performances were observed between the 2 groups, except for a higher water intake for group C. Finally, during the period corresponding to diet F3 (60–95 d) there were only differences in feed intake, which was higher for the AD group. In conclusion, the substitution of soybean meal for *Acheta domesticus* meal caused a decrease in feed intake during the first month and consequently a lower body weight. During the first 4 weeks of life of the chicks, a partial replacement of soybean meal may be recommended, since high inclusions of *A. domesticus* meal in this period seem to be detrimental in young chicks. Given the absence of relevant differences in productive performances between both groups, it could be concluded that the use of *A. domesticus* cricket meal can be a potential protein alternative to soybean meal.

## INTRODUCTION

The world's population is exponentially increasing, estimated at 9.7 billion inhabitants in 2050 (Newbold and Population growth, 2017), and along with it the consumption of resources from the primary sector. This population growth, together with dietary changes toward greater consumption of meat products, has encouraged poultry meat to become an alternative to meet this food demand (Allegretti et al., 2018).

Besides, the increasing reluctance against certain conventional raw materials used in the feed industry, triggered by their negative environmental sustainability, highlights the need to look for new alternative protein sources for animal feed (Abro et al., 2020). Particularly worrying is the case of soybean as the main protein source in animal feed (Vauterin et al., 2021), since its cultivation is one of the main causes of deforestation worldwide, it is associated with an enormous water consumption, an indiscriminate use of pesticides and chemical synthesis products, as well as an irreparable loss of biodiversity, given the monopoly of the transgenic varieties used (Sánchez-Muros et al., 2014). This scenario has motivated a growing interest in nonconventional feed ingredients that are more efficient in terms of production performance and environmental sustainability (de Souza Vilela et al., 2021), especially those that represent an alternative protein source, the price, and availability of which may jeopardize poultry meat production (Nalunga et al., 2021).

Among the alternative sources of animal feed, insect meal is considered a high protein and environmentally sustainable food, since its production is associated with low land and water requirements, low carbon footprint and a circular economy model (Abro et al., 2020). Insect-based protein is rapidly emerging as an alternative food source both as a potential substitute for soy-based protein for use in animal feed and for direct human consumption (Vauterin et al., 2021). The use of insect meal is even more interesting in certain animal husbandry systems. Particularly, it is considered a potential substitute for fishmeal and soybean meal in the diets of conventional poultry systems (Barroso et al., 2014; Khan, 2018; Nieto et al., 2022), due to its high protein content, its rapid production owing to the short life cycle of insects and its relative ease of production (Perera and Bhujel, 2021).

Besides, consumers are increasingly demanding greater transparency in the production chain, in which achieving a sustainable, healthy product that also complies with animal welfare has become a priority objective (Font-i-Furnols and Guerrero, 2022). For this reason, slow-growing chick strains are more and more used in the meat poultry sector, with longer production cycles and a greater adaptability to alternative production systems (Allais et al., 2019; Vissers et al., 2019). Although these strains need more time to reach market weight, their feed conversion might become more efficient when using new ingredients included in their diets, plus presenting nutritional characteristics more appreciated by consumers (Filho et al., 2021).

Furthermore, given that the natural behavior of chicks is to feed on different insects throughout their lives (Khan, 2018), strong preferences of chicks for diets composed of insect meal offered simultaneously with other ingredients have already been revealed (Filho et al., 2020). Therefore, it could be expected that the use of insect meal in their diet would have a positive impact on both the productive performance of chickens and the nutritional quality of their meat. In the scientific literature, some experiences have been reported on the use of more common insect meals in the market, such as the *Tenebrio molitor* or the *Hermetia illucens* (de Marco et al., 2015; Biasato et al., 2017; de Souza Vilela et al., 2021), in shorter production cycles of approximately 2 mo, typical of broilers (Bovera et al., 2016; Nascimento Filho et al., 2021).

In addition, European regulation recently allowed the use of *Acheta domesticus* as an ingredient in monogastric diets, together with *Herrmetia illucens, Musca domestica, Tenebrio molitor, Alphitobius diaperinus, Gryllodes sigillatus*, and *Gryllus assimilis*. Considering the above the main objective of this work was to assess the effect of cricket (*Acheta domesticus*) meal made from insects in the early stages of adulthood (incomplete metamorphosis) on the performance of slow-growing chicks during 95 d of life, acting as a total substitute for soybean meal. Three different diets were used throughout the life of the animals, monitoring their growth along the trial.

## MATERIALS AND METHODS

### Chick Management and Experimental Design

The experiments were conducted in accordance with the principles and specific guidelines presented in the Guide for the Care and Use of Agricultural Animals in Research and Teaching, 4th edition, 2020, avoiding unnecessary discomfort to the animals. Particularly, all slow-growing chicks used in the trial were treated in accordance with the principles of European Parliament and Council Directive 2003/65/EC of 22 July 2003, amending Directive 86/609/EEC regarding the protection of animals used for experimental and other scientific purposes. These animals were manipulated under Order ECC/566/2015, of 20 March, establishing the training requirements to be met by personnel handling animals used, bred or supplied for experimental and other scientific purposes, including teaching. The experimental protocol was approved by the Bioethics Committee of the University of Salamanca (Spain), with registration number 0000590 regulated by RD 53/2013, of 1 February, establishing the basic rules applicable for the protection of animals used in experimentation and other scientific purposes, including teaching. The trial was conducted by qualified personnel from the Animal Production area in the facilities and experimental fields of the University of Salamanca.

A total of 128 *Colorield*-strain one-day-old male slow-growing chicks (hereinafter named as chicks), vaccinated in the hatchery facilities against Marek´s disease and avian infectious bronchitis, were used in this experiment. After their reception, these chicks were randomly separated into 16 different experimental units (n=8 chicks each) on the basis of maintaining a homogeneous initial life weight among them. In turn, 8 of the 16 experimental units were randomly selected to comprise the control group (C) (mean live weight of 36.0 ± 0.8 g), that was fed soybean meal as the main protein source, and the remaining units formed the Acheta group (AD) (mean live weight of 36.5 ± 0.6 g), whose main protein intake was *Acheta domesticus* meal, as a total replacement of soybean meal. All the chicks were raised until 95 d of age and then slaughtered in an authorized slaughterhouse in compliance with Council Regulation (EC) No. 1/2005 of 22 December 2004 on the protection of animals during transport and related operations and amending Directives 64/432/EEC and 93/119/EC and Regulation (EC) No. 1255/97 and with Council Regulation (EC) No. 1099/2009 of 24 September 2009 on the protection of animals at the time of slaughter.

During the first 45 days of life, each experimental unit was housed in a 1.50 × 0.60 m^2^ indoor pen (0.10 m^2^/chick) with first age feeders and drinkers until day 20, which were replaced by second age ones from that day onward. The floor of the pens was cover with 7 cm thick layer of wood shavings. Temperature and humidity were progressively fitted to the optimum life conditions of the chicks in each moment of the experiment. Specifically, the average temperature inside the barn was 31.9 ± 1.8ºC during the first week, 29.1 ± 2.1ºC the second week, 25.4 ± 1.7ºC the third week and 20.8 ± 2.7ºC for the rest of the trial. Overall, the chicks were subjected to natural daily photoperiods (10–11 hours).

From the 46th day of life (approximately half of the cycle of the chicks) until the end of the trial, the 128 chicks were moved to an outdoor facility divided into sixteen 8.2 × 4 m^2^ pens (0.25 m^2^/chick) with plastic mesh, where they had uninterrupted freedom of movement. The soil of the outside yard was completely natural, neither treated nor cultivated, and it was perimetrically enclosed by a 1 m high metal sheet, which was buried 30 cm, and a 13 × 13 mm grid plastic mesh covering roof and sides to prevent predators from entering. Moreover, these pens counted with circular feeders and drinkers and a wooden shelter with a single entrance on the south side (0.5 × 0.5 m^2^), and an upper opening to ensure a good ventilation and a translucent section for lighting.

### Composition of the Experimental Diets and Data Treatment

Three concentrated diets (F1, F2 and F3) were used throughout the whole experiment, feeding the chicks for about a month. Thus, F1 was used from day 1 to day 29, F2 from day 29 to day 60 and F3 from day 60 to day 95. The proportions of the raw materials used in the composition of these diets are shown in Table 1. Regardless the diet used, both groups of chicks (C and AD) were isoenergetically and isoproteically fed throughout the whole experiment. Thus, in order to meet the nutritional requirements of the birds, the recommendations of the National Research Council (NRC, 1994) for medium-slow differentiated growth chicks were followed. Moreover, prior to fed these diets to the chicks, they were chemically and nutritionally analyzed (Table 2) in the food analysis laboratories of the Abiomed Higiene S.L. company (Salamanca, Spain), who operates under an ISO 9001 quality system.

**Table 1:** Composition of the experimental diets for two groups of chicks (% ingredients), fed diets including either soybean meal (group C) or *Acheta domesticus* insect meal (group AD) as the main protein source.

Ingredients	C^1^			AD^2^		
	F1^3^	F2^4^	F3^5^	F1^3^	F2^4^	F3^5^
Corn	37.0	48.0	31.0	0.0	9.0	16.0
Wheat	11.0	12.0	37.0	12.0	20.0	38.0
Barley	0.0	0.0	0.0	32.0	16.0	0.0
Soybean meal	29.0	20.0	12.0	0.0	0.0	0.0
*A. domesticus* meal	0.0	0.0	0.0	17.0	10.0	7.3
Sunflower	19.0	16.0	16.0	0.0	11.0	8.7
Oats	0.0	0.0	0.0	35.0	30.0	26.0
Vitamin-mineral premix^6^	4.0	4.0	4.0	4.0	4.0	4.0

1 C: control group.

2 AD: *Acheta domesticus* group.

3 F1: 1–29 days.

4 F2: 29–60 days.

5 F3: 60–95 days.

6 Vitamin-mineral prefix includes in percentage: Methionine 0.21; Calcium 20.75; Phosphorus 5.58; Sodium 6.38. Includes per kg of diet: Vitamin D3 10,0490 UI, Vitamin E 398 UI, Vitamin A 251,230 UI, Betaine anhydrous 4,002.84 mg. Anti-caking agents: bentonite 10%; sepiolite 3,340.2 mg/kg. Trace element premix includes per kg of diet Cu (sulfate II pentahydrate), 335 mg; Fe (sulfate II monohydrate), 1,324 mg; Mn (oxide), 3,336 mg, Zn (oxide), 2,835; I (calcium iodate anhydrous), 51.07 mg; Se (sodium selenite), 10.10 mg.

**Table 2:** Chemical/nutritional characteristics of the experimental diets for two groups of chicks fed diets including either soybean meal (group C) or *Acheta domesticus* insect meal (group AD) as the main protein source.

	F1 (1–29 d)		F2 (29–60 d)		F3 (60–95 d)			
Item	C^1^	AD^1^	C	AD	C	AD	Soybean meal	*A. Domesticus* meal
%Moisture	10.1	9.1	10.7	9.3	10.2	9.7	12.1	4.4
%Ashes	6.1	6.4	4.8	6.3	5.6	5.9	6.2	4.1
%Crude fat	12.1	8.4	9.8	12.4	6.7	10.7	1.9	22.6
%Crude fiber	6.0	8.3	5.3	8.2	4.7	7.4	5.9	6.9
%Starch	35.8	33.8	45.1	37.1	45.5	41.5	0.1	1.7
%Crude protein	19.8	19.3	15.7	15.2	14.6	14.8	44.0	59.5
ME^2^ (Kcal/kgDM)	2,802.8	2,897.3	2,900.0	2,966.2	2,950.8	2,978.3	1,890.0	5,817.0
Mean dietary crude protein (%)	19.6		15.5		14.7		44.0	59.5
Mean dietary ME (Kcal/kgDM)	2,850.0		2,933.1		2,964.6		1,890.0	5,817.0
%Ca^3^	1.24	1.19	1.21	1.19	1.20	1.18	0.29	0.25
%P^3^	0.40	0.61	0.36	0.50	0.34	0.43	0.61	2.13
%SFA^4^	1.6	2.7	1.3	2.4	0.9	2.1	0.6	5.3
%MUFA^4^	7.9	3.1	6.2	7.6	4.0	6.3	0.9	8.9
%PUFA^4^	2.6	2.6	2.3	2.3	1.9	2.3	2.6	8.5
%n-3 PUFA^4^	0.17	0.09	0.13	0.08	0.11	0.08	0.31	0.27
%n-6 PUFA^4^	2.4	2.5	2.2	2.3	1.79	2.18	2.27	8.20
%Trans FA^5^ (C18:1T+C18:2T+C13:3T)	<0.05	<0.05	<0.05	<0.05	<0.05	<0.05	<0.05	<0.05
Aspartic acid + Asparagine (g/100 gDM)	1.78	1.02	1.62	0.85	1.26	1.10	4.84	3.60
Glutamic acid + Glutamine (g/100 gDM)	3.09	2.36	3.05	3.14	3.01	2.36	7.56	6.30
Alanine (g/100 gDM)	1.19	1.26	0.77	0.87	0.77	0.84	1.89	5.0
Arginine (g/100 gDM)	1.37	0.82	1.36	0.91	1.04	0.85	3.18	3.40
Cysteine (g/100 gDM)	<20	<20	<20	<20	<20	<20	<20	<20
Cystine (g/100 gDM)	0.11	0.11	0.13	0.19	0.14	0.13	0.70	0.24
Phenylalanine (g/100 gDM)	0.91	0.51	0.76	0.50	0.68	0.55	2.19	1.62
Glycine (g/100 gDM)	1.33	1.22	1.19	1.05	0.79	1.03	1.89	2.68
Histidine (g/100 gDM)	0.81	0.44	0.65	0.47	0.52	0.57	1.17	1.46
Isoleucine (g/100 gDM)	0.74	0.46	0.59	0.41	0.51	0.48	1.96	2.03
Leucine (g/100 gDM)	1.50	0.88	1.25	0.82	0.89	0.73	3.29	4.20
Lysine (g/100 gDM)	1.16	0.85	1.08	0.59	0.83	0.65	2.68	2.83
Methionine (g/100 gDM)	0.20	0.17	0.19	0.16	0.19	0.18	0.59	0.69
Proline (g/100 gDM)	1.18	1.09	1.12	0.73	1.07	0.86	2.16	3.70
Serine (g/100 gDM)	1.18	0.75	1.02	0.65	0.90	0.84	2.11	1.70
Tyrosine (g/100 gDM)	0.56	0.46	0.43	0.36	0.36	0.44	1.73	4.40
Threonine (g/100 gDM)	0.76	0.59	0.63	0.46	0.55	0.55	1.72	2.01
Tryptophan (g/100 gDM)	<20	<20	<20	<20	<20	<20	<20	<20
Valine (g/100 gDM)	0.82	0.62	0.64	0.59	0.59	0.63	2.07	3.70

1 C: control group, AD: *Acheta domesticus* group.

2 ME, metabolizable energy.

3 Ca, calcium, P, phosphorous.

4 SFA, saturated fatty acids; MUFA, monounsaturated fatty acids; PUFA, polyunsaturated fatty acids; n-3 PUFA, omega-3 polyunsaturated fatty acid; n-6 PUFA, omega-6 polyunsaturated fatty acid.

5 Trans FA, trans fatty acid; DM, dry matter.

Both water and feed were supplied *ad libitum*, being the latter provided to the chicks in find grind form. Although the health status of the birds was daily monitored, the following variables were weekly measured during the whole trial, according to the procedure indicated in Nieto et al. (2022): live body weight (**LBW**), body weight gain (**BWG**), feed intake (**FI**), water intake (**WI**), and feed conversion ratio (**FCR**).

### Statistical Analysis

As aforementioned, 16 experimental units randomly assigned to 2 different groups were considered in this trial. In order to assess the potential significant differences between C and AD groups for each of the studied variables, a multivariate analysis of variance (**MANOVA**) fitted to a general linear model (**GLM**) was use, taking diet and group as fix factors. The initial weight at the beginning of the second and third stages (d 29 and 60, respectively) were included in the model as covariates, given the heterogeneity of those weights between groups in these specific moments of the trial. Prior to this procedure, the Kolmogorov–Smirnov test was performed to check the normality of the recorded data. The statistical significance was assessed at the 95% confidence level (a = 0.05) using Snedecor's F as the contrast statistic.

Regarding the assessment of the possible existence of differences in the LBW increment (%) between C and AD groups throughout the trial, a Student's *t* test for paired samples was conducted. Contrary, differences in the BWG increment (%) between the C and AD groups for each of the 3 diets in the trial (F1, F2 and F3) were analyzed using a Student's *t* test for independent samples.

All the statistical processing of the data was performed using IBM-SPSS Package 26 software (IBM, Chicago, IL). Results were expressed as mean and pooled standard error of the mean (**SEM**).

## RESULTS

### Productive Performance

Overall, the results suggested similar performances between both experimental groups (C and AD) in all the variables studied throughout the trial, with no significant differences between groups (Table 3). However, there were differences between the performance of the groups within each of the stages of the experiment, corresponding to each of the diets supplied (F1, F2 and F3).

**Table 3:** Total production performance parameters of two chicken groups fed diets including either soybean meal (group C) or *Acheta domesticus* insect meal (group AD) as the main protein source for the three different diets offered (mean ± SEM^1^).

Variable	Group	F1 (1–29 d)	F2 (29–60 d)	F3 (60–95 d)	Total (1–95 d)
LBW^3^^,^^4^ (g)	C^2^	353.71	1,084.74	2,484.26	2,484.26
	AD^2^	291.41	960.25	2,121.05	2,121.05
	SEM^1^	9.80	32.35	72.09	72.09
	*P*-value	0.000	0.762	0.763	0.763
BWG^3^ (g)	C	11.35	24.10	41.31	25.02
	AD	9.10	21.58	33.17	22.18
	SEM^1^	0.35	1.06	1.28	1.18
	*P*-value	0.000	0.948	0.255	0.617
FI^3^ (g)	C	896.90	2,499.40	4,502.85	7,899.15
	AD	766.33	2,826.10	5,276.97	8,869.40
	SEM^1^	22.21	158.36	237.81	346.72
	*P*-value	0.001	0.581	0.030	0.459
WI^3^ (mm)	C	1,245.20	4,005.01	9,305.92	14,556.13
	AD	1,040.87	3,277.89	7,323.75	11,642.51
	SEM^1^	35.04	211.91	621.71	873.91
	*P*-value	0.001	0.010	0.259	0.189
FCR^3^	C	2.83	3.35	3.32	3.39
	AD	3.01	4.24	4.55	4.26
	SEM^1^	0.04	0.31	0.39	0.30
	*P*-value	0.043	0.274	0.207	0.097

1 SEM: pooled standard error of the mean.

2 C: control group, AD: *Acheta domesticus* group.

3 BWG, body weight gain; FCR, feed conversion ratio; FI, feed intake; LBW, live body weight; WI, water intake.

4 LBW was punctually measured on the last day of each period, i.e., on days 29, 60, and 95.

At the end of the stage corresponding to the F1 diet, group C reached a higher LBW than group AD (P=0.000). Consequently, the BWG in this first period was also higher in the chicks of group C compared to those of group AD (P=0.000). Regarding intakes, FI and WI were higher for group C (P=0.001;P=0.001, Table 3). Differences were also observed for the FCR, being higher for the AD group (P=0.043). However, at the end of the diet F2, differences between C and AD groups were only observed for the WI (P=0.010), with higher values for the former (Table 3). In the case of the diet F3, FI presented significant differences between groups (P=0.030), being the chicks of group AD the ones that consumed a higher amount of feed.

The weekly analysis (Table 4) revealed differences in LBW and BWG among the experimental groups (C and AD) within the period of the F1 diet, particularly between weeks 2 and 4 (LBW: $P_{2w}$ = 0.000;$P_{3w}=0.000;P_{4w}=0.000$; BWG: $P_{2w}=0.000;$ $P_{3w}=0.013;P_{4w}=0.001$), with higher values for group C. Furthermore, in this first period, group C chicks showed higher FI from weeks 1 to 3 ($P_{1w}=0.046;P_{2w}=0.000;P_{3w}=0.000$) than the chicks from group AD. As for the WI, group C chicks consumed more water than the group AD chicks during the second and fourth weeks ($P_{2w}=0.001;$ $P_{4w}=0.000$;). However, the FCR was higher for group C during the first week, but lower during the second and fourth weeks than the FCR of the AD group ($P_{1w}=0.034;P_{2w}=0.003;P_{4w}=0.000$). During the F2 diet period, differences between experimental groups were found regarding for LBW and WI (Table 4). Specifically, the chicks of group C were heavier than the chicks of group AD at the end of the eighth week ($P_{8w}=0.003$), which corresponded to the measure taken on d 53. During the ninth week, chicks from group C consumed more water than the ones from group AD ($P_{9w}=0.021$). Contrary to the results found within the previous diets, during the period of the diet F3·there were not differences between the 2 experimental groups regarding growing performance (Table 4), albeit differences in the WI were exhibited in the last week of the trial ($P_{14w}=0.028$), with a higher consume, again, for the chicks of group C.

**Table 4:** Weekly production performance parameters of the chicken groups fed diets including either soybean meal (group C) or *Acheta domesticus* insect meal (group AD) as the main protein source. for the three different diets offered (mean ± SEM^1^).

Variable	Group	Indoor stage							Outdoor stage						
		F1 (1–29 d)				F2 (29–60 d)					F3 (60–95 d)				
		1 w (1–8 d)	2 w (8–15 d)	3 w (15–22 d)	4 w (22–29 d)	5 w (29–36 d)	6 w (36–43 d)	7 w^5^ (43-46 d)	8 w (46–53 d)	9 w (53–60 d)	10 w (60–67 d)	11 w (67–74 d)	12 w (74–81 d)	13 w (81–88 d)	14 w (88–95 d)
LBW^3^^,^^4^ (g)	C^2^	60.84	113.29	207.66	353.71	518.82	699.26	776.58	906.32	1,084.74	1,339.21	1,628.95	1,950.26	2,242.89	2,484.26
	AD^2^	61.95	95.08	174.78	291.41	426.17	569.31	630.66	768.25	960.25	1,162.75	1,409.00	1,656.25	1,899.00	2,121.05
	SEM^1^	0.86	2.73	5.34	9.80	13.73	18.81	21.11	28.39	32.35	43.01	51.74	58.36	65.98	72.09
	*P*-value	0.478	0.000	0.000	0.000	0.095	0.134	0.191	0.003	0.762	0.650	0.702	0.785	0.807	0.763
BWG^3^^,^^4^ (g/day)	C	3.55	7.49	13.48	20.87	23.59	25.78	25.78	22.07	23.36	36.35	41.39	45.90	41.80	34.48
	AD	3.63	4.73	11.39	16.66	19.25	20.45	20.10	21.23	27.43	28.93	35.18	35.32	34.68	31.72
	SEM^1^	0.15	0.39	0.43	0.72	0.67	0.83	0.85	1.25	3.26	2.20	1.47	1.58	1.56	1.39
	*P*-value	0.479	0.000	0.013	0.001	0.094	0.506	0.506	0.983	0.059	0.650	0.851	0.254	0.949	0.519
FI^3^^,^^4^ (g)	C	21.55	34.15	35.94	36.50	58.21	74.02	80.18	82.50	107.97	92.37	120.88	137.97	144.16	147.90
	AD	18.63	25.75	27.90	37.19	54.11	68.10	71.71	94.50	156.29	150.89	126.07	138.90	161.97	176.04
	SEM^1^	0.71	1.24	1.24	0.75	1.16	1.50	1.79	7.93	19.55	23.74	10.31	2.31	6.58	11.92
	*P*-value	0.046	0.000	0.000	0.761	0.152	0.302	0.348	0.789	0.285	0.243	0.078	0.726	0.234	0.466
WI^3^^,^^4^ (mm)	C	22.42	32.14	52.98	70.35	97.92	107.82	132.26	128.50	181.22	179.17	216.00	267.65	296.92	369.69
	AD	22.61	26.82	47.38	51.88	67.57	82.17	92.49	113.61	165.29	166.79	194.36	207.18	205.18	272.75
	SEM^1^	0.38	1.01	1.69	2.85	4.75	3.79	6.02	10.17	4.78	10.56	8.77	19.95	29.26	28.06
	*P*-value	0.789	0.001	0.124	0.000	0.750	0.415	0.050	0.114	0.021	0.887	0.520	0.317	0.261	0.028
FCR^3^^,^^4^	C	6.13	4.58	2.68	1.75	2.47	2.88	3.12	3.60	4.35	2.60	3.09	3.18	3.63	4.61
	AD	5.27	5.52	2.48	2.25	2.81	3.35	3.52	4.50	5.79	5.21	3.56	3.94	4.71	5.53
	SEM^1^	0.26	0.18	0.07	0.07	0.06	0.09	0.08	0.34	0.50	0.93	0.32	0.26	0.40	0.41
	*P*-value	0.034	0.003	0.173	0.000	0.207	0.852	0.690	0.694	0.146	0.175	0.260	0.393	0.420	0.582

1 SEM: pooled standard error of the mean.

2 C: control group, AD: *Acheta domesticus* group.

3 BWG, body weight gain; FI, feed intake; FCR, feed conversion ratio; LBW, live body weight; WI, water intake.

4 LBW was punctually measured on the last day of each period, i.e., on days 8, 15, 22, 29, 36, 43, 46, 53, 60, 67, 74, 81, 88, and 95.

5 7 w (43–46 d) is composed only of four days since it is the point in which the chicks were carried to the outdoor stage.

### Assessment of the Growth and the Feed Intake

**Figure 1:**
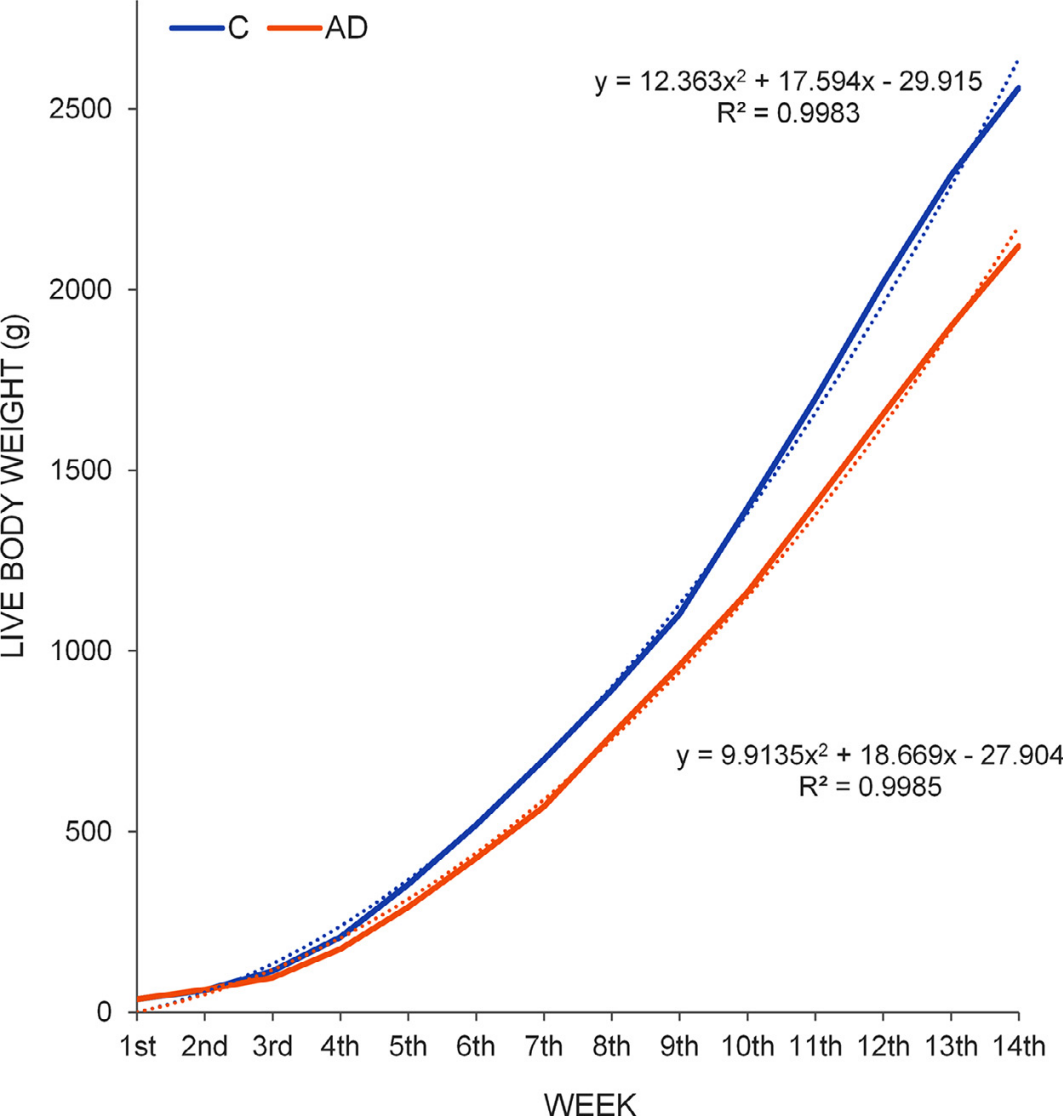
LBW curves for two groups of chicks fed diets that included soybean meal (group C) or *Acheta domesticus* insect meal (group AD) as the main protein source throughout the growing period (14 wk). Discontinuous lines reflect degree 2 polynomial trend lines of the two LBW curves, whose equations and goodness-of-fit (R^2^) are also shown.

**Figure 2:**
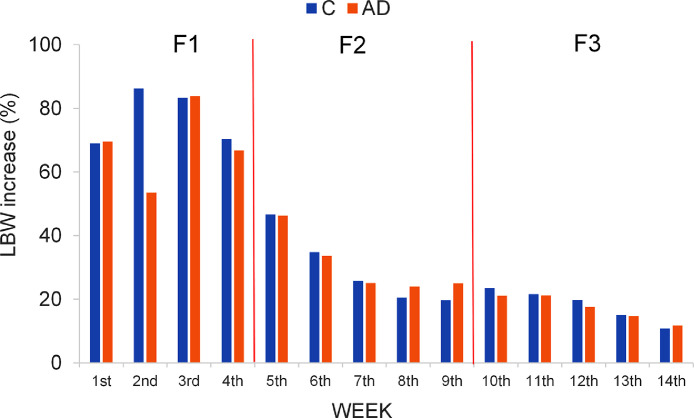
LBW increase (%) for two groups of chicks fed diets that included soybean meal (group C) or *Acheta domesticus* insect meal (group AD) as the main protein source between two consecutive weeks over the entire studied period.

**Figure 3:**
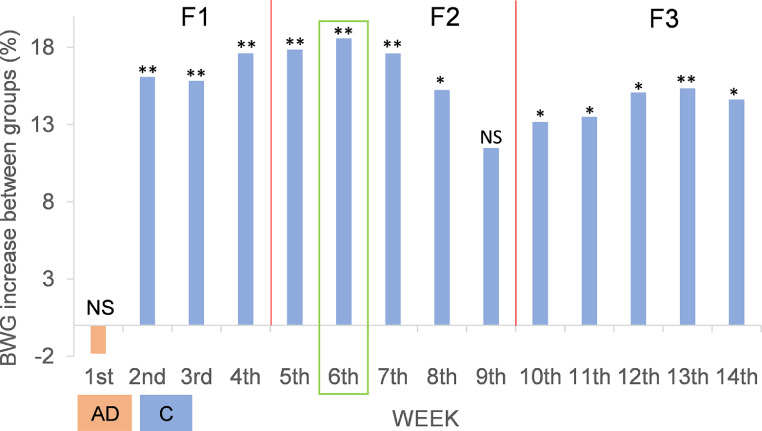
BWG increase (%) for two groups of chicks fed diets that included soybean meal (group C) relative to the group fed *Acheta domesticus* insect meal (group AD) as the main protein source over the entire period studied. NS, * or **: nonsignificant or significant at *P* < 0.05 or 0.01, respectively.

The results suggested that the LBW curves of both experimental groups fit almost perfectly to grade 2 polynomial trend line (Figure 1), based on the R^2^ values. Furthermore, from these results can be stated that the chicks from group AD grew slower than the chicks from group C from the second week until the end of the trial. The LBW increase of the birds, expressed as a percentage, was different among consecutive weeks (P=0.001) throughout the whole experiment, being higher in the first stages of their life (Figure 2). The separate analysis of each group revealed that the greatest increase in LBW for group C corresponded to the second week; in contrast to the AD group that occurred in the days of the third week. Differences were found in the growth rate increase, expressed in terms of BWG, between the C and AD groups from the second week of life to the end of the trial (Figure 3), except at week 9, with higher increases for the C group compared to the AD group. The largest difference between the 2 groups occurred at week 6 of life (green rectangle of Figure 3).

## DISCUSSION

The results obtained in this study revealed that the inclusion of a high percentage of insect meal in the diet of slow-growing chicks during their first month of life negatively affects their productive performance, contrary to what occurs from the second month onward. These results agree with those of Nieto et al. (2022) in their work with *Tenebrio molitor* meal, but contrast with those found by Nakagaki et al. (1987), who showed no difference in the BWG of the birds caused by the substitution of soybean meal for cricket meal during the first 14 days of life.

During the period corresponding to the F1 diet, there was a decrease in feed intake by the AD group, along with a lower final weight. The feeding of both experimental groups was slightly different in terms of fat, calcium and fiber content, the first 2 being lower in the diet of the AD group, while fiber content was higher. Some authors (Makkar et al., 2014) also found that diets containing insect meal as a protein source are often deficient in calcium. Possibly, the proportion of *Acheta domesticus* during their first days of life may affect their digestibility, as Kovitvadhi et al. (2019) found, proving that crude protein from the meal of different crickets (including *Acheta domesticus*) showed a worse *in vitro* digestibility than soybean and fish meal.

During the feeding of diets F2 and F3 there were hardly any differences in the productive performance of the chicks, except in the case of water consumption in F2 and feed consumption in F3. These results coincide with those described by Finke et al. (1985), in which the inclusion of crickets in broiler diets to replace soybean meal showed no differences in final weights or in the feed/weight gain ratio, corroborating the feasibility of their use. In the case of the work of Bovera et al. (2015), the use of insects in isoproteic and isoenergetic diets as a substitute for soybean did not affect consumption during the second month of life of chickens. Biasato et al. (2016) did not find differences in feed conversion after the inclusion of insect meal in broilers between 43 and 97 d of age. Similarly, Leiber et al. (2015) found no differences in feed intake or body weight gain between broilers that consumed insect and those that did not between 7 and 82 d of age. Therefore, the substitution of soybean meal by insect meal appears to be a valuable feed management with good production rates in alternative poultry systems (DeFoliart et al., 1982; Wang et al., 2004; Khan et al., 2018; Benzertiha et al., 2020; Kim et al., 2020).

In the light of these results, it could be stated that including high concentrations of house cricket meal (>10%) in the diet of chickens during the first stages of their life might be detrimental to the birds, since it disinhibits the chickens to consume the feed and, therefore, negatively affects their BWG (Atteh and Ologbenla, 1993; Bamgbosei, 1999). In fact, Benzertiha et al. (2020) found a worse FCR in those chicks that consumed insect meal in high concentrations during the first 35 d of life. In the same line, Perera and Bhujel (2021) used cricket meal to replace fish meal in *Oreochromis niloticus* diets, obtaining better results in growth with partial substitutions than with total substitution. Similarly, Wang et al. (2005) argued that the use of cricket meal in isoproteic and isonergic poultry diets was more productive and beneficial at low inclusion levels. This result could be extrapolated to other insect species, as it is the case of *Tenebrio molitor* larval meal (Biasato et al., 2017, 2018; Nieto et al., 2022).

Therefore, insect meals could partially replace soybean products in poultry diets without affecting feed efficiency (Leiber et al., 2015). However, it has been shown that total substitution at early stages is inappropriate given the production losses involved (Nieto et al., 2022). This could be explained by the hard outer chitin layer of insects, which is difficult to digest by domestic birds (Ravindran and Blair, 1993), especially in the early stages of life when the digestive system of chicks is very immature. Therefore, soybean meal (or other alternative protein source) should continue to be part of the diets of the chicks in the first stage, being its total substitution feasible from the first 4 weeks of life, as proposed in the work of Olkowski (2018). In addition, the results obtained in the trial showed that the initial differences between groups (C and AD) decreased as the trial progressed, that is, as the content of the insect in the diet decreased, ultimately improving the performances of the chicks that consumed *Acheta domesticus* meal.

In conclusion, this study assessed the effect of totally replacing soybean meal with *Acheta domesticus* meal as the main protein source in isoprotein and isoenergetic diets of slow-growing male *Colorield* chicks during their entire productive cycle. The results obtained suggested that it is not appropriate to include high levels of insect meal in the diet of chicks during the first 4 weeks of life. Instead, a partial substitution of soybean meal could be recommended, since high amounts of insect meal seem to be detrimental in young chicks. Therefore, under these conditions, *Acheta domesticus* meal may be an interesting protein source to include in slow-growing chicks as a partial substitute for soybean meal during the first month of birds' life and as a full replacement from that time onward until the end of the productive cycle.
